# Association of Longitudinal Nutrient Patterns with Body Composition in Black Middle-Aged South African Women: A Five-Year Follow-Up Study

**DOI:** 10.3390/ijerph191912792

**Published:** 2022-10-06

**Authors:** Caroline B. T. Makura-Kankwende, Philippe J. Gradidge, Nigel J. Crowther, Tshifhiwa Ratshikombo, Julia H. Goedecke, Lisa K. Micklesfield, Shane A. Norris, Tinashe Chikowore

**Affiliations:** 1 SAMRC/Wits Developmental Pathways for Health Research Unit (DPHRU), Faculty of Health Sciences, University of the Witwatersrand, Johannesburg 2198, South Africa; 2 Department of Exercise Science and Sports Medicine, Faculty of Health Sciences, University of the Witwatersrand, Johannesburg 2198, South Africa; 3 Department of Chemical Pathology, National Health Laboratory Service, Faculty of Health Sciences, University of the Witwatersrand, Johannesburg 2192, South Africa; 4 Biomedical Research and Innovation Platform, South African Medical Research Council, Cape Town 7505, South Africa; 5 University of Stirling, Stirling FK9 4LA, UK; 6 School of Human Development and Health, University of Southampton, Southampton SO17 1BJ, UK

**Keywords:** nutrient patterns, body composition, adiposity, African women

## Abstract

This study aimed to evaluate the association of longitudinal nutrient patterns with body composition in a cohort of 132 black South African middle-aged women over five years. Nutrient patterns were identified using principal component analysis at baseline and follow-up 5 years later. Associations between nutrient patterns and repeated body composition measures were evaluated using generalized estimating equations, before and after adjusting for baseline education and repeated measures of age, socio-economic status, physical activity and employment. The animal-driven nutrient pattern was associated with increases in repeated measures of visceral adipose tissue (VAT) (β coefficient, 5.79 [95% CI, 0.01–11.57] cm^2^), fat mass index (FMI) (0.47 [0.01–0.93] kg·m^−2^) and lean mass index (LMI) (0.50 [0.18–1.17] kg·m^−2^) (*p* < 0.05) after adjustment. Vitamin C, sugar, and potassium-driven nutrient pattern was associated with higher FMI (0.50 [0.12–0.88] kg·m^−2^) and LMI (0.58 [0.07–1.10] kg·m^−2^) before and after adjustment (*p* < 0.05). These findings suggest that dietary interventions to curb obesity in black middle-aged South African women should focus on attenuation of nutrient patterns centred on added sugar, animal fat and animal protein.

## 1. Introduction

Metabolic syndrome, high blood pressure, and type 2 diabetes are all more common in black women, who have higher rates of obesity, than in white women, according to research conducted in South Africa [1,2]. In South Africa, it has been estimated that NCDs account for ~51% of all fatalities with ~19% of all deaths attributable to cardiovascular diseases [3]. Additionally, this increased risk of NCD onset may be due to black women showing a unique body composition phenotype by storing more subcutaneous adipose tissue (SAT) than other ethnic groups [4]. Furthermore, it has been demonstrated that ageing in black women is characterized by significant increases in visceral adipose tissues (VAT) and a relative redistribution of fat from the peripheral to the central region [4].

Obesity has been shown to have strong links with dietary behaviours [5,6,7]. As a result, designing health promotion programmes that focus on dietary behaviours is critical [8,9]. To create appropriate and effective health promotion programs, information on long-term nutritional behaviours and associations with health outcomes is required. 

The United Nations’ Food and Agriculture Organization (FAO) and the World Health Organization (WHO) have formed a joint consultation to develop national food-based dietary guidelines (FBDGs), which provide context-specific advice and principles on healthy diets based on scientific evidence [10]. This has resulted in the FAO/WHO 2004 Global approach on food, physical activity, and the amended 2012 South African FBDGs, among other recommendations and reports [10]. These dietary guidelines are regularly updated with the most relevant information and therefore there is a need for frequent nutritional studies to provide both country and population specific results to inform such guidelines. 

The body composition of women has also been proven to be significantly influenced by their race or ethnicity, and numerous cross-sectional studies have documented these racial disparities in body composition and associations with nutrient patterns. Nutrient patterns of middle-aged black South African women have not been studied longitudinally to date. There have only been five cross-sectional studies examining the nutrient patterns of adult men and women aged 18 and older in South Africa [11,12,13,14,15]. These cross-sectional studies unfortunately are unable to show sustainability of a populations nutrient patterns while longitudinal nutrient pattern studies results show sustainability of nutrient patterns in the population and longitudinal association of adiposity, therefore, appropriate long-term nutritional advice can be given. Of these studies, two documented specific nutrient patterns in black South African women, the same cohort for this study, and evaluated the association between these nutrient patterns and adiposity with the one study looking at sex differences in the associations of nutrient patterns with total and regional adiposity [11,15]. This study showed that a plant driven nutrient pattern explained the greatest variance in nutrient intake in this population and was positively associated with SAT [11]. This study aims to investigate changes in body composition over 5.5 years, and to determine if these changes are attributable to nutrient patterns extracted at baseline and follow-up. 

## 2. Materials and Methods

### 2.1. Study Population and Setting 

**Figure 1:**
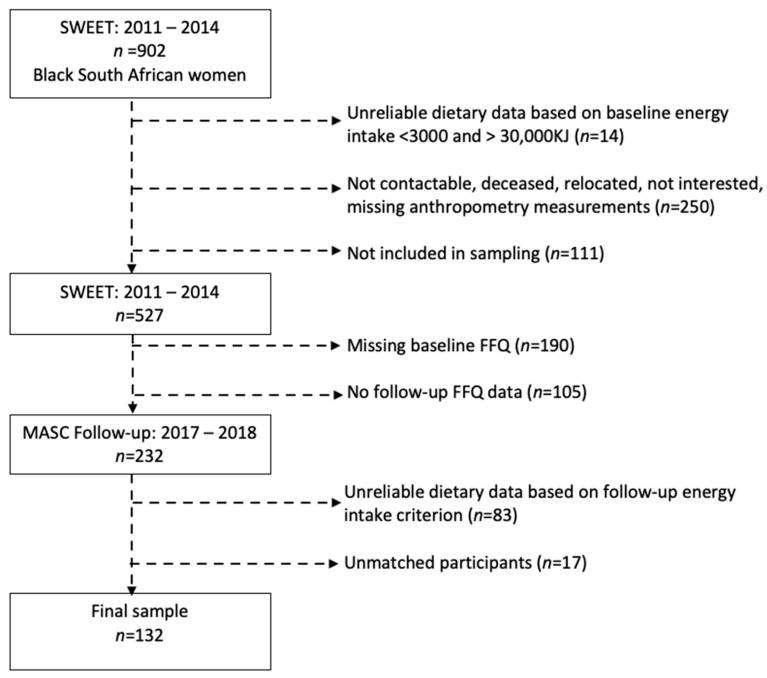
Flow chart of study participants. (SWEET = Study of Women in and Entering Endocrine Transition; MASC = Middle-aged Soweto Cohort).

At baseline, a total of 902 women who were the biological mothers of the Birth to Twenty Plus (BT20) cohort were invited to participate in the Study of Women in and Entering Endocrine Transition (SWEET) study [11]. The following inclusion criteria were applied: between 40 and 60 years of age, not pregnant, black African women (Figure 1) [16,17]. Between January 2017 and August 2018, 527 women from the SWEET cohort were randomly sampled and invited to participate in a follow-up study which was subsequently referred to as the Middle-aged Soweto Cohort (MASC) [18]. After excluding participants that were not contactable, deceased, relocated, not interested, missing anthropometry measurements or not included in sampling, did not have baseline nutrition data, or were a mismatched participant, the final sample size of women with dual-energy X-ray absorptiometry (DXA) body composition measurements and dietary data at baseline and follow-up was *n* = 132 (Figure 1). The study was conducted according to the guidelines of the Declaration of Helsinki and approved by the Human Research Ethics Committee (Medical) of the University of the Witwatersrand (ethics numbers: M160604 and M160975).

### 2.2. Dietary Intake

Dietary intake data was collected at baseline and follow-up using a standardised quantitative food frequency questionnaire (QFFQ), designed for the South African population [11,19,20,21,22]. The QFFQ consists of 214 food items that are representative of foods consumed by most of the South African population currently [23]. Although this tool was tested in adolescents, it has since been piloted and utilised extensively in the general population [11,20,21,22]. Trained interviewers used high quality photographs of food items to assist with participant recall of food and beverage items consumed during the last seven days. In the case of food items consumed in the last seven days, additional data on the frequency and quantity of consumption were collected. The participants were asked to estimate their habitual intake by selecting the most accurate representation of their portion size from either two-dimensional actual size drawings of foods, household utensils, or three-dimensional validated food models as described by Steyn et al. [23]. These household measures were then converted to grams for the computation of average intake over a seven-day period. Food items were then converted to nutrients using the nutrient analysis software FoodFinder3 developed and hosted by The South African Medical Research Council [24]. Participants with unreliable dietary data based on energy intake <3000 and >30,000 kJ, as described by Vorster et al. [25], were excluded (*n* = 20 with either unreliable dietary data at baseline (*n* = 14) or follow-up (*n* = 6)).

Baseline and follow-up plausibility of energy intake reporting was adjusted in Generalized estimating equations and this variable was calculated using the ratio of the dietary energy intake (EI) and estimated energy requirements (EER) which is the average dietary energy intake that is predicted to maintain energy balance in healthy, normal weight individuals of a defined age, gender, weight, height, and level of physical activity consistent with good health [11,26]. The EER was calculated using the Institute of Medicine (IOM) gender-specific equations shown below for females: 

EER = 354 − ((6.91 × Age (years)) + Physical Activity (PA) coefficient (9.36 × Weight (kgs)) + 726 × Height (m))

This equation considers the participant’s age, weight, height, and standard pre-defined physical activity (PA) coefficient from the equation, which is gender and age specific and classified according to different ranges of physical activity levels. For adult women reporting a low active lifestyle, the PA coefficient is 1.12 as moderate intensity physical activity has been reported for this same population which is in line with findings from this study as well [27]. Participants with EI:EER ratio less than 0.7 were classified as under-reporters, 0.7 to 1.42 as plausible reporters, and greater than 1.42 as over-reporters. Plausible energy intake was used as the reference category in statistical models for the EI: EER ratio [26].

### 2.3. Body Composition Measurements

Participants were requested to remove their shoes and to wear lightweight clothing prior to weigh in. Weight was recorded to the nearest 0.01 kg. Height was measured and recorded to the nearest 0.1 cm. Body mass index (BMI) was calculated as weight (in kilograms) divided by the square of baseline height (in metres).

A single trained technician performed dual-energy X-ray Absorptiometry (DXA) scans at baseline and follow-up using a Hologic Discovery software version 12.1 (Hologic Inc., Bedford, MA, USA, APEX software). Whole-body scans were completed to determine whole body (minus head) fat mass (FM) and whole-body lean mass (fat-free soft-tissue mass). The coefficient of variation (CVs) for the DXA parameters were <2% for total fat mass and 1% for fat-free soft tissue mass. Fat mass was used to calculate fat mass index (FMI) (fat mass (kg)/baseline height (m^2^)), and lean mass was used to estimate lean mass index (LMI) (whole-body lean mass (kg)/ baseline height (m^2^)) [28]. Visceral adipose tissue (VAT), subcutaneous adipose tissue (SAT) and gynoid fat were also estimated using DXA [29].

### 2.4. Socio-Demographics and Physical Activity 

Details of socio-demographic and lifestyle characteristics were collected at baseline and at follow-up and questionnaires were administered by research assistants. Socio-demographic factors included age, self-reported employment status (Yes/No) and highest education level achieved (completed primary school, incomplete high school and completed high school). Household socio-economic status was estimated using a count of the following major household amenities: electricity, radio, motor vehicle, fridge, washing machine, mobile, microwave, electronic media network (MNET) and digital satellite television. 

Self-reported physical activity was collected using the Global Physical Activity Questionnaire (GPAQ) [30]. Participants were categorised as active or inactive at baseline and follow-up according to the GPAQ criteria [30]. Participants were categorised as active if they reported >600 MET minutes per week [31]. Participants who did not meet these criteria were classified as inactive. Physical activity was further categorized into three groups: active participants at both time points, inactive at both time points, and those who changed their physical activity in any direction between the time points (spectrum of physical activity). 

### 2.5. Statistical Analysis: Analysis of Longitudinal Data

All statistical analyses were performed using STATA version 15 (StataCorp, College Station, TX, USA) and IBM SPSS version 27 software packages (IBM, Chicago, IL, USA) [32,33]. Q-Q plots were used to perform normality tests on the continuous variables and normally distributed continuous variables are presented as mean ± SD, while non-parametric data are presented as median (interquartile range [IQR]) for the descriptive tables. The Student paired *t*-test, Chi-square test or the Wilcoxon signed rank test were used to explore differences between baseline and follow-up measurements for normally distributed continuous data, categorical data, and not normally distributed continuous data, respectively.

Principal component analysis (PCA) was applied at both baseline and follow-up to extract nutrient patterns from 25 nutrients derived from the QFFQ [34]. Total available carbohydrates were divided into starch, total sugar and dietary fibre. The nutrients were log transformed to remove bias due to variance as a result of the different measures of scale used to quantify the nutrients [35]. As previously described, the PCA approach was performed using the covariance matrix of the log transformed nutrients that were selected to capture the entire dietary intake [11,13,14]. Varimax rotation was used to make the factor loadings more understandable [36]. The top three principal components (PCs) indicating three distinct nutrient patterns were selected and plotted on a circular plot based on their eigenvalues and visual appearance on the scree-plot of eigenvalues and the proportion of total variance explained. Nutrient patterns at baseline and follow-up were used as predictor variables.

Generalized estimating equation (GEE) analyses were used to analyse longitudinal associations of both baseline and follow-up measures of nutrient patterns with six repeated measures of body composition: BMI, VAT, SAT, FMI, LMI and gynoid fat. The GEE analysis extends the generalized linear model to allow for the analysis of repeated measurements. It combines within-subject relationships with between-subjects relationships into a single regression coefficient [37,38]. Furthermore, GEE allows for the explicit modelling of a wide range of correlation structures and is more interpretable than repeated measures analysis of variance (ANOVA) [37,38]. The GEE models examined time-varying nutrient patterns while accounting for the correlation of repeated measures for the same participant. Two GEE models were constructed, GEE model 1 was adjusted for all nutrient patterns and energy intake reporting and GEE model 2 was adjusted for the same variables as model 1 plus the following: baseline education and repeated measures of age, household socio-economic status (SES), physical activity levels and employment. Furthermore, the GEE models for the regional adiposity measures (VAT, SAT, and gynoid fat mass) were further adjusted for FMI to explore the effects of regional adiposity as opposed to total adiposity. The β coefficients and the 95% confidence interval (CI) from GEE models were reported as the measures of association.

## 3. Results

### 3.1. Descriptive Characteristics

The characteristics of the participants are summarized in Table 1. Despite no significant changes in BMI, LMI and SAT, DXA-derived measures of fat mass, FMI, VAT and gynoid fat mass increased over the 5-year follow-up while lean mass decreased. At baseline and follow-up, over 65% of the women were classified as having overweight or obesity.

Total energy intake significantly decreased over the 5 years (from 9.3 mJ to 6.4 mJ, respectively) although the proportion of carbohydrates and protein consumed remained stable across the two time points, while fat consumption significantly increased over the 5 years. Whole-body fat mass, fat mass index, gynoid fat mass and visceral adipose tissue all increased at follow-up (*p* value < 0.05) while whole body lean mass decreased at follow-up (*p* value = 0.029). 

### 3.2. Nutrient Patterns 

**Figure 2:**
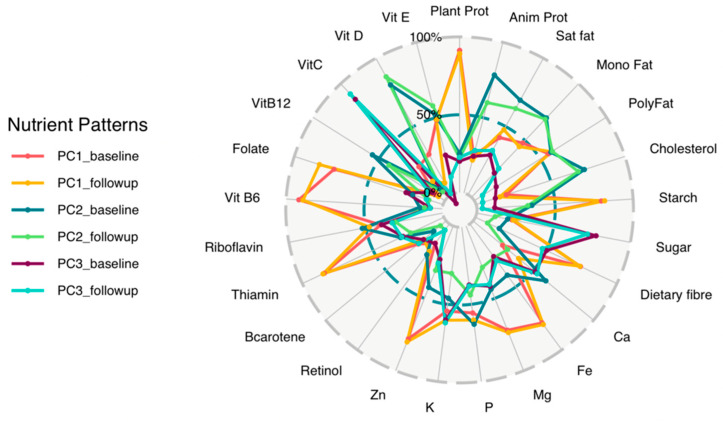
Baseline and follow-up PC derived nutrient patterns. This is based on 25 nutrient items in middle-aged black South African women (*n* = 132). Identified by principle component analysis, on the circular plot, the first 3 PCs that met the Scree test which was used to determine the number of PCs to retain and eigenvalues are shown by different coloured lines (referred to as PCA nutrient pattern 1–3) for both baseline and follow-up and each component represents an independent nutrient pattern. PC = principal component; Plant Prot = plant protein; Anim prot = animal protein; Sat fat = saturated fat; Mono Fat = monounsaturated fat; Poly Fat = polyunsaturated fat; Ca = Calcium; Fe = Iron; Mg = magnesium; P = phosphorus; K = potassium; Zn = zinc; Vit B6 = vitamin B6; Vit B12 = vitamin B12; Vit C = vitamin C; Vit D = vitamin D; Vit E = vitamin E; PC1 = plant driven nutrient pattern; PC2 = animal protein driven nutrient pattern; PC3 = vitamin C, sugar and potassium driven nutrient pattern. The orange and yellow line (PC1) represents the factor loadings related to the plant driven nutrient pattern (baseline and follow-up, respectively), the dark and light green lines (PC2) represents factor loadings related to the animal protein driven nutrient pattern (baseline and follow-up) and the purple and light blue lines (PC3) represents factor loadings related to the vitamin C, sugar and potassium driven nutrient pattern (baseline and follow-up).

Baseline and follow-up nutrient patterns were extracted using principal component analysis (PCA) and results of the nutrient patterns displayed as a radar plot and a table (Figure 2 and Table S1 respectively). 

These nutrient patterns were identified and driven by the same nutrients at baseline and follow-up (Figure 2). These nutrient patterns explained 69% and 64% of the total variation of nutrients consumed by the study participants at baseline and follow-up, respectively. Although the value of the factor loadings of the individual nutrients changed between baseline and follow-up, the nutrients with the highest loadings for each principal component (PC) did not change therefore the nutrient patterns remained the same. The first nutrient pattern (PC1) was the “Plant driven nutrient pattern” with high factor loadings of plant protein, starch, and B vitamins, followed by the “Animal protein driven nutrient pattern” (PC2) characterised by high factor loadings of animal protein and saturated fat, and the third nutrient pattern (PC3) was the “Vitamin C, sugar and potassium driven nutrient pattern” characterised by high factor loadings of sugar, vitamin C and potassium. 

### 3.3. Longitudinal Association between Nutrient Patterns and Body Composition Indices 

Univariate analysis using GEE and individual body composition measures showed that the associations of the plant based nutrient pattern with FMI and LMI were significant, however these associations were no longer significant after adjusting for covariates in multivariate analysis (Table 2).

The animal protein driven nutrient pattern was associated with significant increases in FMI, LMI and VAT for model 1 and this remained significant after adjustment (note, the GEE VAT model was further adjusted with FMI). Repeated measures of the vitamin C, sugar, and potassium driven nutrient pattern was significantly associated with an increase in FMI and LMI before and after controlling for household SES and related covariates. None of the nutrient patterns were associated with BMI, SAT and gynoid fat when adjusted for all the model covariates (the animal protein nutrient pattern was significantly associated with increases in gynoid fat in unadjusted model). 

## 4. Discussion

In this study, we explored the longitudinal relationship between nutrient patterns and various measures of body composition in black middle-aged South African women. Three nutrient patterns were extracted at baseline and follow-up which were plant driven, animal protein driven and vitamin C, sugar, and potassium driven nutrient pattern. The identified nutrient patterns described 69% and 64% of the total variation in nutrient consumption at baseline and follow-up, respectively. Repeated measures of VAT, FMI and LMI were positively associated with repeated measures of the animal-driven nutrient pattern, while repeated measures of FMI and LMI was positively associated with repeated measures of the vitamin C, sugar, and potassium-driven nutrient pattern. The plant driven nutrient pattern was associated with repeated measures of FMI and LMI, however, after adjusting for co-variates, significance was lost.

The nutrient patterns of adult black South Africans have previously been studied in 4 cross-sectional studies [11,12,13,15]. Chikowore et al. examined associations between nutrient patterns and fasting glucose and glycated haemoglobin levels while Conradie et al. examined the quality of pregnant women diet. Makura-Kankwende et al. and Ratshikombo et al., both cross sectional studies, examined associations of nutrient patterns with body composition measurements with the later study aimed at examining the association between nutrient patterns and DXA-derived body fat and regional adiposity in middle-aged black South African men and women and determine if this differed by sex. The patterns extracted from these previous studies are comparable to those found in our study despite minor discrepancies in the nomenclature of the patterns and suggests that these nutrient patterns are common and consistent in the black South African population. 

There were no significant changes in BMI, only significant changes in DXA-derived measures namely fat mass, FMI, VAT, lean mass and gynoid were observed over the 5-year follow-up period. Despite the paucity of research on the longitudinal effects of nutrient patterns on adiposity, our findings are consistent with other cross-sectional studies that show that the animal protein driven nutrient pattern, was positively associated with repeated measures of FMI and VAT, measures of total and central body fat, respectively [6,39,40]. Makura-Kankwende et al., Rosqvist et al. and Fischer et al. showed that saturated fats or animal protein driven diet were associated with an increase in VAT in adults which is in line with findings from this study [39,40]. The storage of animal fat as triglycerides in adipose tissue resulting in increased adiposity is thought to explain the association of animal protein driven nutrient patterns with increased VAT and FMI [6,39]. These changes to total and central body fat may be attributable to ageing, which, in turn, may contribute to increased risk of development or progression of NCDs in LMIC. This may result in over-burdened health care facilities and limited resources thus impacting the management of NCDs. 

Our investigation also found that consumption of the animal protein driven nutrient pattern was associated with an increase in LMI. These findings are similar to other longitudinal studies that also reported that nutrients from animal food sources resulted in increases in lean body mass [41,42,43]. Lean mass and adiposity indices were positively associated with animal protein intake in the Osteoporosis Risk Factor and Fracture Prevention Study (OSTPRE-FPS) [42]. Furthermore, a meta-analysis study which included 18 studies found that a diet high in animal protein was associated with increased lean mass compared a diet high in plant protein which supports the findings from this study [43]. It is thought that the consumption of animal protein may cause the activation of protein synthesis leading to an increase in lean mass [7,44]. This suggests that a diet high in animal protein is advantageous for lean mass growth however, this nutrient pattern was also associated with higher fat mass therefore recommendations are needed on how to leverage this nutrient pattern to promote lean mass and not increase fat mass. The variance of the animal protein driven nutrient pattern which relates to consumption, was far less than the plant driven nutrient pattern. 

Our findings showed that a vitamin C, sugar, and potassium driven nutrient pattern was associated with higher FMI and LMI. Foods and beverages that are rich in these nutrients include starchy vegetables such as white potatoes and sweet potatoes and sugar-sweetened beverages for example which may contribute to increased adiposity, while foods such as green leafy vegetables such as spinach may lead to increased LMI [45]. This nutrient pattern has a wide diverse range of food which includes both beneficial and harmful food choices, therefore further research is needed to help identify which foods are beneficial. Observational studies have revealed that high potassium intake may be associated with increased lean mass, along with other body composition changes, consistent with our findings where the vitamin C, sugar and potassium driven nutrient pattern had higher factor loadings for potassium [46,47]. In addition, results from a longitudinal study conducted in adult men and women over a 3-year follow up period showed that a higher intake of foods rich in potassium, supported preservation of lean mass [48]. It is suggested that potassium preserves lean mass by neutralising an acidic environment which promotes protein-energy wasting and loss of lean mass [48]. Other epidemiological research has found that high consumption of foods high in vitamin C, have been linked to increased fat mass, possibly due to higher sugar levels [49,50,51]. Dietary sugar may be converted to fat and stored in the body which might explain the association of the sugar-based nutrient pattern with FMI in this and other studies [49,50,51]. 

Dietary assessment studies are often hampered by inconsistencies in the self-reporting of food intake, which we worked to minimise by correcting for energy intake reporting. The study population underreported their energy intake more noticeably during the second visit compared to the first visit and these could be due to consumption of more recent food items that were not consumed at baseline but are now consumed at follow-up that might not be recorded on the QFFQ. Despite these limitations, to our knowledge, this is the first longitudinal study of nutrient patterns and body composition conducted in black African women which provides evidence on consistent, stable nutrient patterns of the population compared to a cross sectional nutrient pattern study. Furthermore, our investigation used DXA-derived measurements of body composition which provide more accurate assessments of body composition where we were able to examine associations between nutrient pattern and both lean mass and fat mass. Previous studies used simple anthropometric body composition measurements. Finally, while the study was focused on black South African middle-aged women, gender and age specific studies should be conducted to investigate whether the same nutrient patterns are dominate in men or the younger population and if the nutritional recommendations can be applied across the various age and gender groups.

## 5. Conclusions

This study documented the nutrient patterns of black South African middle-aged women and indicated that these patterns were sustained over 5 years of follow-up. The consumption of animal based nutrient patterns and vitamin C, sugar, and potassium based nutrient patterns were associated with both increased adiposity and lean mass. More importantly, we found that animal protein was associated with visceral adiposity which is a major risk factor of cardiometabolic diseases. This is in line with recent data from large cohorts that confirmed that total and animal proteins are associated with the risk of cardiovascular disease. Additional research including larger sample sizes and a longer follow-up time is needed to verify these results.

## Figures and Tables

**Table 1:** Baseline and follow-up descriptive characteristics (*n* = 132).

	Baseline	Follow-Up	*p* Value
Demographics			
Age, years	48 (44; 52)	53 (50; 58)	<0.001
Socio-economic status (a score out of 9)	5 (4; 6)	5 (3; 5)	0.572
Physically active (%)	94 (71.2%)	93 (70.5%)	0.882
Body composition			
BMI (kg m^−2^)	33.7 (28.1; 39.0)	33.7 (28.1; 39.2)	0.767
Whole-body fat mass (kg)	60.5 (47.1; 73.3)	63.4 (49.1; 76.3)	0.015
Fat mass index (FMI) (kg m^−2^)	23.5 (20.5; 30.3)	26.6 (20.7; 31.8)	<0.001
Whole body lean mass (kg)	41.9 (36.9; 45.1)	41.0 (34.4; 52.4)	0.029
Lean mass index (LMI) (kg m^−2^)	16.6 (14.7; 18.2)	16.4 (14.5; 20.8)	0.055
Gynoid fat mass (kg)	3.2. (2.6; 4.2)	3.6 (3.0; 4.6)	<0.001
Subcutaneous Adipose Tissue (cm^2^)	452.7 (360.2; 574.5)	457.6 (349.1; 576.4)	0.547
Visceral Adipose Tissue (cm^2^)	102.6 (75.2; 128.9)	107.8 (70.4; 137.3)	0.001
Nutritional intake data			
Energy intake (mJ)	9.3 (7.9; 12.0)	6.4 (5.1; 8.4)	<0.001
% Carbohydrates (of total energy)	54.5 (49.7; 58.1)	52.6 (48.6; 56.5)	0.052
% Protein (of total energy)	11.4 (10.1; 12.8)	11.2 (9.7; 12.7)	0.871
% Fat (of total energy)	30.1 (26.0; 34.3)	31.7 (27.0; 35.5)	0.035

**Table 2:** GEE β coefficients for repeated measures of nutrient patterns and repeated measures of body composition (*n* = 132).

	BMI		VAT		SAT		FMI		LMI		GYNOID	
	Model 1	Model 2	Model 1	Model 2	Model 1	Model 2	Model 1	Model 2	Model 1	Model 2	Model 1	Model 2
Plant Protein Driven NP	0.138	0.290	0.952	−4.040	−1.432	−2.135	0.461 **	0.361	−0.890 **	0.150	0.127	−0.787
Animal Protein Driven NP	0.307	0.243	10.115 ***	5.788 **	1.451	−7.103	0.449 ***	0.469 **	0.466 *	0.498 **	0.724 **	0.002
Vit C, Sugar, and Potassium Driven NP	0.157	0.404	3.525	0.010	6.096	−2.785	0.425 ***	0.502 ***	0.499 **	0.584 **	0.308	−0.622

Note: *** p < 0.01, ** p < 0.05, * p < 0.1. Bold values denote statistical significance at *p* < 0.05. Model 1: Adjusted for all NPs and energy intake reporting. Model 2: Adjusted for M1 plus employment, baseline education, energy intake reporting, physical activity, socioeconomic score. * FMI included only for VAT, SAT and gynoid GEE models.

## Data Availability

The data that support the findings of this study are available from the corresponding author upon reasonable request.

## Supplementary Materials

The following supporting information can be downloaded at: https://www.mdpi.com/article/10.3390/ijerph191912792/s1, Table S1: Baseline and follow-up extracted nutrient patterns and factor loadings for the Nutrient Patterns for the nutrient patterns.
