# Supplementary material for: Association of Longitudinal Nutrient Patterns with Body Composition in Black Middle-Aged South African Women: A Five-Year Follow-Up Study
Source: Int J Environ Res Public Health. 2022 Oct 6;19(19):12792. doi: 10.3390/ijerph191912792 (PMC9565998; doi:10.3390/ijerph191912792)
Supplement: Supplementary file 1 [file ijerph-19-12792-s001.zip › ijerph-1914703-supplementary.pdf]

Table S1: Baseline and follow-up extracted nutrient patterns and factor loadings for the Nutrient Patterns for the nutrient patterns

	Baseline			Follow-up		
	Plant Driven Nutrient Pattern	Animal Driven and Potassium Nutrient Pattern	Vitamin C, Sugar Driven Nutrient Pattern	Plant Driven Nutrient Pattern	Animal protein driven Nutrient Pattern	Vitamin C, Sugar and Potassium Driven Nutrient Pattern
**Plant Protein**	**0.913**	0.250	0.202	**0.892**	0.222	0.226
**Animal Protein**	0.248	**0.783**	0.244	0.219	**0.600**	0.282
**Saturated fat**	0.419	**0.693**	0.291	0.476	**0.632**	0.324
**Monounsaturated Fat**	0.478	**0.699**	0.213	0.446	**0.688**	0.254
**Polyunsaturated Fat**	0.562	0.580	0.166	0.586	0.591	0.062
**Cholesterol**	0.203	**0.727**	0.129	0.159	**0.686**	0.044
**Starch**	**0.797**	0.352	0.114	**0.818**	0.326	0.035
**Sugar**	0.229	0.202	**0.777**	0.239	0.120	**0.721**
**Dietary fibre**	**0.743**	0.169	0.502	**0.739**	0.086	0.474
**Calcium**	0.245	**0.604**	0.516	0.378	0.288	0.532
**Iron**	**0.786**	0.407	0.259	**0.799**	0.266	0.291
**Magnesium**	**0.720**	0.436	0.416	**0.736**	0.285	0.404
**Phosphorus**	0.556	**0.629**	0.375	**0.602**	0.440	0.381
**Potassium**	0.544	0.461	**0.601**	**0.603**	0.300	**0.619**
**Zinc**	**0.780**	0.424	0.231	**0.801**	0.307	0.256
**Retinol**	0.153	0.245	0.155	0.218	0.048	0.051
**Beta carotene**	0.233	0.103	0.188	0.186	0.048	0.228
**Thiamin**	**0.845**	0.306	0.289	**0.854**	0.235	0.297
**Riboflavin**	0.403	0.522	0.397	0.477	0.305	0.328
**Vitamin B6**	**0.919**	0.144	0.077	**0.892**	0.114	0.077
**Folate**	**0.733**	0.205	0.249	**0.831**	0.095	0.112
**Vitamin B12**	0.073	0.549	0.095	0.049	0.424	0.132
**Vitamin C**	0.269	0.074	**0.863**	0.141	0.048	**0.910**
**Vitamin D**	0.296	**0.805**	-0.065	0.086	**0.865**	0.009
**Vitamin E**	0.483	0.520	0.252	0.488	0.579	0.109
**Explained variance %**	32.157	23.748	13.577	33.583	17.133	13.212
**Cumulative var % explained**	32.157	55.904	69.482	33.583	50.716	63.928

Bold factor loadings indicate factor loadings >±0.600 which were used for naming the nutrient patterns
